# Clinical characteristics and imprinting analysis of Chinese Silver Russell Syndrome

**DOI:** 10.1186/1687-9856-2015-S1-P108

**Published:** 2015-04-28

**Authors:** Di Wu, Chunxiu Gong, Yang Zhao

**Affiliations:** 1 Beijing Children’s Hospital, Capital Medical University, Beijing, China

## Aims

To study clinical characteristics and imprinting defects in Chinese children with SRS.

## Methods

Forty-nine SRS cases were studied retrospectively. Out of these 49 cases, 36 were available to be detected chromosome 11p15 imprinting defects and 21 cases were detected uniparental disomy of maternal chromosome 7 (UPD[7] mat).

## Results

There were 32 boys and 17 girls whose ages ranged from 3 m to 12 y. The main clinical characteristics of these SRS were: i) SGA and postnatal growth retardation (mean height standard deviation score (HT SDS) was 2.25; ii) Skeletal malformation including triangular-shaped face, small chin, irregular/crowded teeth, limbs asymmetry and fifth finger clinodactyly. Genetic analysis showed that ICR1 hypomethylation were 22/36 (61.1%) which were following: Ten had hypomethylation in chromosome 11p15 imprinting control region 1 (ICR1) of the paternal allele; seven had both hypomethylation in ICR1 and ICR2; five had hypomethylation in ICR1 and hypermethylation in ICR2. And UPD7 (mat) positive is 1/21 (4.8%). Six patients had been treated with growth hormone (GH) for 3 to 24 months. Growth rates ranged from 4 to 10.8 cm/year.

## Conclusions

This study demonstrated that Chinese children with SRS had more growth retardation than bone retardation and had classical skeletal malformation such as triangular faces, and limb asymmetry. Chromosome 11p15 imprinting defects contributed to over 60% of these cases and UPD7 (mat) positive is 4.8%.

